# The hunter or the hunted? Inferring benthic meso-predator behavior from swim velocity data

**DOI:** 10.7717/peerj.21782

**Published:** 2026-10-06

**Authors:** Perla Salzeri, John P.Y. Arnould

**Affiliations:** School of Life and Environmental Sciences, Faculty of Science, Engineering and Built Environment, Deakin University, Melbourne, Victoria, Australia

**Keywords:** Pinnipeds, Marine mammals, Behavioural ecology, Seascape of fear, Predator, Meso-predator, Benthic dives, Fur seals, Animal behaviour

## Abstract

**Background:**

Air-breathing benthic predators must balance foraging with exposure risk under strict oxygen constraints. Animal-borne video data from Australian fur seals (*Arctocephalus pusillus doriferus*; AUFS) has documented prolonged immobility on the sea-floor, but the prevalence, organization, and functional significance of this behavior remain unclear.

**Methods:**

Swim velocity and dive-behavior data from adult female AUFS were analyzed to identify Sea-floor Stationary Dives (SFSD), defined as dives in which ≥50% of the bottom phase occurred at zero swim speed. Temporal variation was assessed using Generalized Additive Models with individual identity as a random effect. Sequential organization was evaluated using run-length distributions and permutation tests (10,000 randomizations preserving each individual’s dive-type totals). Spatial recurrence was examined qualitatively using locations from two GPS-instrumented females. Dive-cycle characteristics associated with SFSD were compared with other benthic dive types.

**Results:**

SFSD comprised ∼2% of all benthic dives and showed pronounced crepuscular peaks with substantial individual heterogeneity. Sequential analyses revealed multi-dive SFSD bouts in a subset of individuals, with significant clustering relative to random expectations. SFSD were associated with intermediate bottom times and short surface intervals, indicating compressed dive cycles. Qualitative spatial patterns from two Global Positioning System (GPS)-instrumented females suggested recurrence of SFSD within localized areas across multiple foraging trips.

**Conclusions:**

The timing, clustering, and dive-cycle characteristics of SFSD suggest this behavior is not associated primarily with ambush predation or resting. Instead, they are most consistent with a predator-avoidance response by air-breathing divers vulnerable in an open sea-scape. These findings highlight the importance of predation risk in shaping dive organization and habitat use in benthic marine predators.

## Introduction

Predator–prey interactions are fundamental drivers of population dynamics, community structure, and ecosystem functioning (Li & Montagnes, 2015; Pettorelli et al., 2011; Schmitz, 2017). Predation outcomes reflect the interaction between how predators move, how prey respond, and the structure of the habitat, creating spatially and temporally uneven risk (Heithaus & Frid, 2003; Wilson et al., 2020). Predators typically rely on either pursuit or ambush, while prey respond with escape, vigilance, crypsis, or refuge use, responses often shaped by habitat complexity (Bedore, Kajiura & Johnsen, 2015; Ioannou & Krause, 2009; Miller, Ament & Schmitz, 2014; Wilson et al., 2018). Furthermore, risk-sensitive behavior changes with body size and predator–prey size ratios, with smaller individuals exhibiting stronger avoidance and larger ones using bolder strategies as vulnerability declines (Emmerson & Raffaelli, 2004; Lima & Dill, 1990). These dynamics create spatially and temporally structured risk landscapes that can shape fine-scale movement and habitat use.

For air-breathing marine predators, foraging behaviors are constrained by dive physiology. Finite oxygen stores require allocation of limited underwater time for descent, search, prey interaction, and ascent, followed by surface recovery (Hindell, 2008). Consequently, the structure of individual dives is shaped by strict aerobic and energetic limits (Hückstädt et al., 2016; Villegas-Amtmann & Costa, 2010; Weise & Costa, 2007). Under such constraints, increases in vigilance and predator avoidance behavior reduce time available for active search and prey capture, imposing temporal trade-offs that influence habitat use and dive organization (Dunphy-Daly et al., 2010).

Benthic foragers experience additional constraints arising from the spatial and temporal distribution of seafloor prey (Fais et al., 2015; Hazen et al., 2011; Womble, Sigler & Willson, 2009). Prey on the seafloor are mostly sedentary and cryptic but distributed in patches associated with substrate characteristics, facilitating predictability in prey location while demanding targeted and often extended search during the bottom phase of dives (Arranz et al., 2011; Hines et al., 2009; Neiße et al., 2020). Benthic foraging is commonly characterized by deeper and longer dives than other marine hunting strategies, constraining the temporal structure of foraging activities and recovery (Belanger et al., 2012; Carlsen et al., 2023; McHuron et al., 2016). Under these circumstances, allocation of time and effort during the bottom phase must balance prey encounter rates with exposure risk, consistent with optimal foraging and risk allocation principles (Ferrari, Sih & Chivers, 2009; Higginson et al., 2012; Pyke, 1984).

The Australian fur seal (*Arctocephalus pusillus doriferus*; hereafter AUFS), the largest fur seal species, breeds in south-eastern Australia where it represents the largest resident marine predator biomass (Kirkman & Arnould, 2018; Kirkwood & Arnould, 2012). Adult females predominantly undertake benthic dives to exploit demersal fish, cephalopods, and elasmobranchs (Arnould & Hindell, 2001; Deagle, Kirkwood & Jarman, 2009; Hoskins et al., 2017). During the approximately 10 month lactation period (Arnould & Hindell, 2001), adult females regularly return to natal colonies for offspring provisioning, imposing central place constraints on foraging (Orians, 1979). These traits restrict the temporal window available for hunting and are reflected in dive profiles dominated by rapid descents, focused bottom phase activity, and short surface recoveries (Salzeri, Luque & Arnould, 2024). As large benthic foragers that are also potential prey, AUFS may experience habitat use and diving decisions that reflect more than foraging alone.

Indeed, animal-borne video recordings from AUFS have documented instances of immobility on the seafloor for extended periods, with some events followed by brief bursts of speed culminating in prey captures (Video S1) and others showing prolonged scanning of the water column before ascent (Video S2). Comparable low movement behavior at depth has been reported in other marine species and interpreted as ambush predation, vigilance, or resting (Briceño et al., 2018; Gayford & Rummer, 2025; Kendall-Bar, 2022). Given the strong costs of immobility for air-breathing divers, understanding the ecological and energetic consequences of stationary seafloor behavior is important because prolonged immobility could shift time budgets away from active prey search, alter habitat-use, and impose opportunity costs that affect offspring provisioning and individual energy budgets (Altshuler et al., 2015; Costa, 2009; Costa & Favilla, 2023; Costa & Gales, 2000). Unfortunately, the small available sample size in AUFS video-recordings limits robust interpretation of the prevalence, timing, and organization of stationary seafloor behavior.

Swim velocity/dive-behavior data loggers have previously been used to investigate foraging behavior, energy expenditure and scaling of stroke frequency in pinnipeds (Carter et al., 2016). In addition, deployments of swim velocity/dive-behavior data loggers can be used to assess seafloor behavior in dives undertaken by benthic foraging individuals (Chung, Lee & Lee, 2021). This enables the occurrence of immobile periods on the seafloor to be assessed across large temporal and spatial scales. Therefore, using swim velocity/dive-behavior data loggers, the aims of the present study were to: (1) quantify the prevalence of stationary seafloor phases within benthic dives; (2) compare the characteristics of stationary seafloor dives with other benthic dives to assess their potential function; and (3) assess the influence of body size, temporal variation and spatial distribution on the frequency of stationary seafloor dives.

## Materials & Methods

### Ethics statement

All data collection involving animals was approved by the Animal Welfare Committees of The University of Melbourne (01146) and Deakin University (A33/2004, A16/2008, A14/2011). Fieldwork was conducted under Wildlife Research Permits issued by the Victorian Department of Sustainability and Environment and the Department of Environment, Land, Water and Planning (Melbourne, VIC, Australia) (permit numbers 10000187, 10000706, 10001143, 10001672, 10005362, 10005848, 10005484, 10007153, and 1000826). Access to Kanowna Island within Wilsons Promontory Marine National Park was granted under permit from Parks Victoria.

### Animal handling and instrumentation

Adult female AUFS provisioning pups at Kanowna Island (39°10′S, 146°18′E) (Fig. 1) were selected at random between April-August from 2002 to 2009 as part of concurrent studies investigating their foraging ecology (Hoskins et al., 2015; Hoskins & Arnould, 2013; Volpov et al., 2016). Captures were conducted using a modified hoop net (Fuhrman Diversified, Seabrook, Texas, USA) and, to facilitate handling, animals were anesthetized with isoflurane delivered *via* a portable vaporizer (Stinger, Advanced Anaesthesia Specialists, Gladesville, NSW, Australia). Each individual was positioned on a restraint board for weighing with a suspension scale (±0.5 kg), and standard morphometric measurements were taken (±0.5 cm). Each AUFS was equipped with a swim velocity/dive-behavior data logger (MK08-V or MK10-V; Wildlife Computers Ltd., Redmond, WA, USA) and a VHF transmitter (Sirtrack Ltd., Havelock North, New Zealand) to facilitate relocation for recapture. Two individuals were additionally equipped with a GPS data logger (F1G; Sirtrack Ltd., Havelock North, New Zealand). Devices were affixed to the dorsal pelage between the scapulae using a two-part epoxy resin (RS Components, Corby, UK). A numbered plastic tag (Super Tags; Dalton, Woolgoolga, Australia) was inserted into the trailing edge of each fore flipper for individual identification. Individuals were allowed to recover from anesthesia and resume normal behaviors. Females were recaptured after completing one or more foraging trips using the same capture protocol. Devices were removed by trimming the fur beneath the attachment site, after which AUFS were immediately released. Total handling time for each animal was less than 30 min.

**Figure 1:**
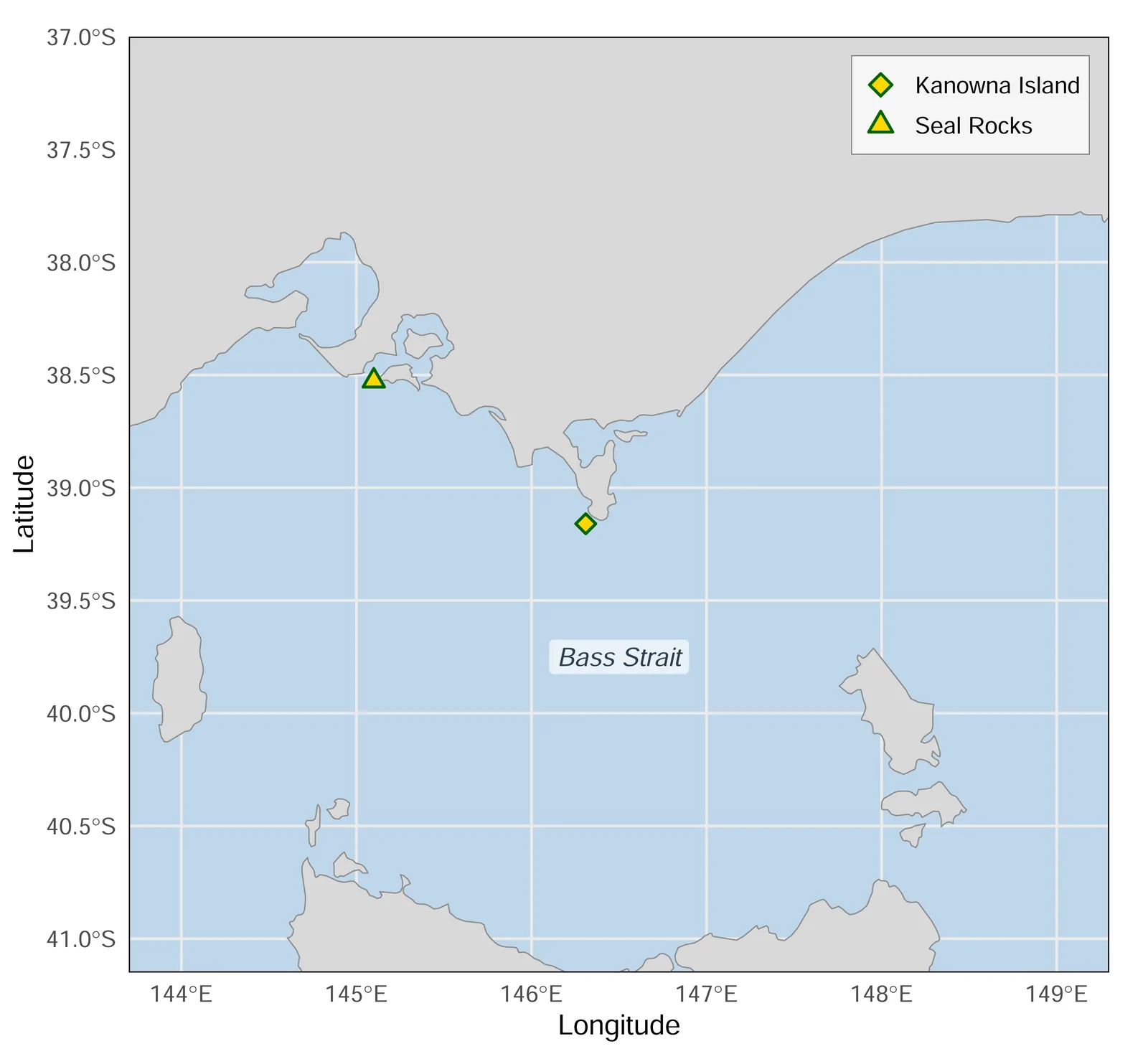
Study area in Bass Strait (south-eastern Australia) showing the locations of Kanowna Island and Seal Rocks. The map shows the Victorian coastline and surrounding waters with latitude/longitude grid, “Bass Strait” is labeled for reference. Kanowna Island is represented as a diamond (39°10′S, 146°18′E), Seal Rocks as a triangle (38°31′S, 145°05′E). Source data and map background: Natural Earth (https://www.naturalearthdata.com/).

### Data processing and dive classification

Data loggers recorded depth and swim velocity every 5 s. The *diveMove* package (Luque, 2007) for the R statistical environment (v4.5.1; R Core Team) was used to zero-offset correct the dive depth data and subsequently process it to extract standard dive metrics, including start time, duration, bottom duration, maximum depth, and post-dive duration. Swim speed during the bottom phase of dives was derived from the onboard speed sensor. Descent and ascent rates were calculated as the vertical distance (m) travelled divided by the duration of the corresponding phase (s). Only benthic dives were retained for analysis (Tremblay & Cherel, 2000). Dives with post-dive durations exceeding 10 min were excluded to remove surface resting periods (Arnould & Hindell, 2001). Next descent rate was defined as the descent rate of the dive immediately following a focal dive, provided the post-dive duration of the previous dive was ≤10 min. Foraging trips were identified from the dive time series as continuous in water intervals >6 h, with trips affected by gaps in the velocity record due to sensor malfunction excluded from analysis.

Benthic dives were classified to identify stationary seafloor phase behavior and post-stationary movement. Sea-floor Stationary Dives (SFSD) were defined as dives in which at least 50% of the bottom phase occurred at swim speed of 0 m s^−1^ (Boyd, Reid & Bevan, 1995). Sensitivity analyses using 25%, 50% criteria showed minimal differences, therefore 50% threshold was adopted for subsequent analyses. Within SFSD, Ambush Indicative Dives (AID) were identified when speed exceeded 1.0 m s^−1^ immediately after the seafloor stationary period, a swim speed increase associated with prey capture events (Salzeri, Luque & Arnould, 2024). The characteristics of SFSD and AID were then compared to two additional dive categories to contextualize these dive types within established benthic behaviors and to test whether they represented distinct dive profiles. Attempted Prey Capture Dives (APCD) were defined as dives with descent rates >1.5 m s^−1^ and no stationary behavior during the bottom phase (Volpov et al., 2016) while Other Benthic Dives (OBD) comprised of all remaining benthic dives. Since APCD were differentiated using descent rate, all comparisons were conducted using dive metrics independent of the classification process (*i.e.,* ascent rate, bottom duration, post-dive duration, and next descent rate).

### Statistical analysis

All analyses were conducted in the R statistical environment (v 4.5.1; R Core Team) with a significance threshold of *α* = 0.05. Dive metrics were compared between dive types using Generalized Additive Models (GAMs, *mgcv* package (Wood, 2025)), fitted separately for each metric (bottom time, post-dive duration, ascent rate, next descent rate). Dive type was the main predictor, and individual was included as a random intercept to account for differences between individuals. From each fitted GAM, estimated marginal means and 95% confidence intervals for each dive type were obtained using the *emmeans* package (Lenth et al., 2019). For Gamma models, estimates were extracted directly on the response scale, whereas for Gaussian models fitted to log-transformed rates, estimates were back-transformed to the original units. Pairwise comparisons among dive types were then conducted using Tukey-adjusted contrasts within each metric.

Dive periods were examined to assess temporal clustering of dive types. Runs were defined as uninterrupted sequences of a given dive type. Because OBD represent a heterogeneous category defined by exclusion and lack a consistent behavioral characteristic, they were omitted from this analysis. APCD were used as a benchmark for patch use clustering as AUFS commonly re descend rapidly and feed repeatedly within productive areas, generating clustered dive sequences (Hoskins et al., 2015; Salzeri, Luque & Arnould, 2024; Volpov et al., 2016). Therefore, clustering in SFSD and AID were evaluated relative to this APCD reference to investigate the extent of their diving periods. For each individual, run lengths were computed across the full dive series and their distributions summarized. To estimate whether observed clustering exceeded random expectation, 10,000 permutations per individual were generated in which the total counts of each dive type were preserved and their order randomized. Run length distributions were recomputed for each permutation. Empirical *p*-values were calculated as the proportion of permuted mean run lengths equal to or exceeding the observed mean run length.

To assess whether body size influenced dive-type proportions, GAMs were fitted with body mass and standard length (SLEN) at device deployment tested separately as individual-level covariates in binomial models of hourly proportions of SFSD and AID among benthic dives (ASM, 1967). Body mass and SLEN were evaluated in separate models because they were strongly correlated, and *p*-values for body-size terms were adjusted using the Benjamini–Hochberg false discovery rate.

Temporal variation in SFSD and AID frequency was also analyzed using GAMs. Hour of day (0–23 h) was modeled using a cyclic cubic spline to enforce 24 h periodicity, and individual identity was included as a random effect to account for repeated measures. Smoothing parameters were estimated by restricted maximum likelihood with penalized splines. Model adequacy was evaluated using Akaike’s information criterion (AIC) and inspection of Pearson residuals.

The spatial distribution of SFSD was investigated in two individuals for which matching Global Positioning System (GPS) tracking data were available. The GPS positions were linearly interpolated at 10 s intervals, and foraging trips were delineated from the time series. For each dive, a location was assigned by matching the dive start time to the nearest interpolated GPS fix. As GPS data were available for only two individuals, descriptive maps of SFSD and other benthic dives were produced but no formal statistical analyses were conducted.

## Results

Trips with gaps in the velocity data due to sensor malfunction were excluded. Continuous sensor data were available for 12 adult female AUFS across 59 complete foraging trips, with 37,802 benthic dives (Table 1; Table S1). Dives with ≥50% of the bottom phase at 0 m s^−1^ comprised 801 dives (2.1% of all benthic dives). Of these, 104 dives (0.3% of all; 13.0% of dives with ≥50% of the bottom phase at 0 m s^−1^) exhibiting a spike in speed (≥1.0 m s^−1^) following seafloor stationary period were classified as AID and 697 dives (1.8% of benthic dives) as SFSD. A further 9,616 dives (25.4% of benthic dives) met the rapid descent criterion for APCD, and the remaining 27,385 dives (72.4% of benthic dives) were grouped as OBD. Total benthic dive counts varied primarily with trip duration (27–217 h per trip, median = 102.2 h, interquartile range (IQR) 73.3–145.6 h) and trip number, such that longer trips or individuals with more numerous trips contributed more dives (per trip counts 153–1,510, median = 601, IQR 373–838; Table S1).

**Table 1:** Summary of trip effort and benthic dive types for 12 individual adult female Australian fur seals (*Arctocephalus pusillus doriferus*). Columns report AUFS ID (Seal), deployment date (dd/mm/yy), body mass (Mass, kg), standard length (SLEN, cm), number of foraging trips (Trips, *n*), total time at sea (h), mean trip duration (h; mean ± SE), total benthic dives (Benthic dives, *n*), number of Sea floor Stationary Dives (SFSD, *n*), proportion of benthic dives classified as SFSD (%), proportion of trip duration spent performing SFSD (%), number of Ambush Indicative Dives (AID, *n*), and proportion of benthic dives classified as AID (%). SFSD were defined as dives with ≥50% of the bottom phase at zero swim speed. AID were identified from swim speed signatures indicative of rapid strike behavior (≥1.0 m s^−^^1^). Dive-type proportions (% benthic dives) were calculated as (count ÷ total benthic dives) × 100. SFSD proportion trip duration (%) was calculated as (time in SFSD ÷ trip duration) × 100.

Seal	Deployment date (dd/mm/yy)	Mass (Kg)	SLEN (cm)	Trips (*n*)	Total time at sea (h)	Mean trip duration (h) Mean ± SE	Benthic dives (*n*)	SFSD (*n*)	SFSD proportion of benthic dives (%)	SFSD proportion trips duration (%)	AID (*n*)	AID proportion of benthic dives (%)
1	26/06/03	45	133	10	1027.48	102.75 ± 15.50	7,661	103	1.34	3.49	10	0.13
2	12/08/03	82.5	158	8	1183.64	147.95 ± 16.86	6,873	6	0.09	0.05	1	0.01
3	08/07/04	76.5	151	1	102.52	102.52	548	3	0.55	0.83	0	0
4	29/04/07	82	161.5	12	1042.49	86.987 ± 4.93	5,751	482	8.38	8.35	76	1.32
5	22/05/08	86.5	155	1	146.52	146.52	341	3	0.88	0.88	0	0
6	25/07/08	71	149	1	110.69	110.69	770	3	0.39	10.01	0	0
7	25/05/08	64.5	138	3	196.45	65.48 ± 27.37	811	12	1.48	0.71	0	0
8	21/05/09	79.5	158	2	177.42	88.71 ± 31.59	1,351	17	1.26	2.21	3	0.22
9	22/05/09	65	151.5	2	133.13	63.25 ± 16.38	974	18	1.85	10.06	0	0
10	23/05/09	91.5	159	1	76.30	76.30	381	2	0.52	0.22	0	0
11	29/05/02	–	165	9	1143.76	118.13 ± 22.61	5,310	39	0.73	1.65	13	0.24
12	29/05/02	–	159	9	1048.13	116.46 ± 20.12	7,031	9	0.13	0.06	1	0.01

All individuals performed SFSD, but prevalence was generally low and variable, with the proportion of benthic dives as SFSD per individual ranging from 0.09% to 8.38% (median 0.81%; IQR 0.46–1.41%). Across individuals, AID were infrequent, with their proportion of dives ranging 0–1.32% (median 0.01%, IQR 0–0.18%) between individuals. Across trips, SFSD represented only a small proportion of at-sea time, with per trip proportions typically below 2–3% (range: 0–26.42%; median 0.32%, IQR 0–3.33%). Aggregated across trips, the total time spent in SFSD clusters per individual was generally modest, ranging from 0.05% to 10.06% of the foraging trips being interrupted by stationary seafloor behavior (median 1.27%, IQR 0.47–5.92%), with some individuals showing concentrated periods where SFSD accounts for a substantial portion of time (Table 1). At the trip level, SFSD occurred in 44 of 59 trips, and AID occurred in 17 of 59 trips (Table S1).

Dive type effects were evident across all dive cycle metrics after accounting for consistent differences among individuals (Fig. 2; Table 2). GAMs fitted separately for each metric, with dive type as the fixed effect and individual included as a random intercept, indicated the strongest separation among dive types for bottom time and next descent rate (Tukey-adjusted pairwise contrasts; Table S2), with more moderate but still detectable differences in ascent rate and post-dive duration. Across metrics, GAMs supported a significant effect of dive type after accounting for among individual variation (Table S2). Deviance explained was modest (∼9–18%), which is not unusual for observational ecological behavioral data from free-ranging animals, where substantial residual variability is expected and environmental covariates that may also influence the response are often not fully captured in the models (Potts & Rose, 2018). Individual identity was included as a random intercept to account for repeated observations within animals, with among-individual variance estimates of 0.0223 (95% CI [0.0096–0.0516]) for bottom time, 0.0362 (0.0158–0.0830) for post-dive duration, 0.00210 (0.00090–0.00486) for ascent rate, and 0.000878 (0.000377–0.00205) for next descent rate.

**Figure 2:**
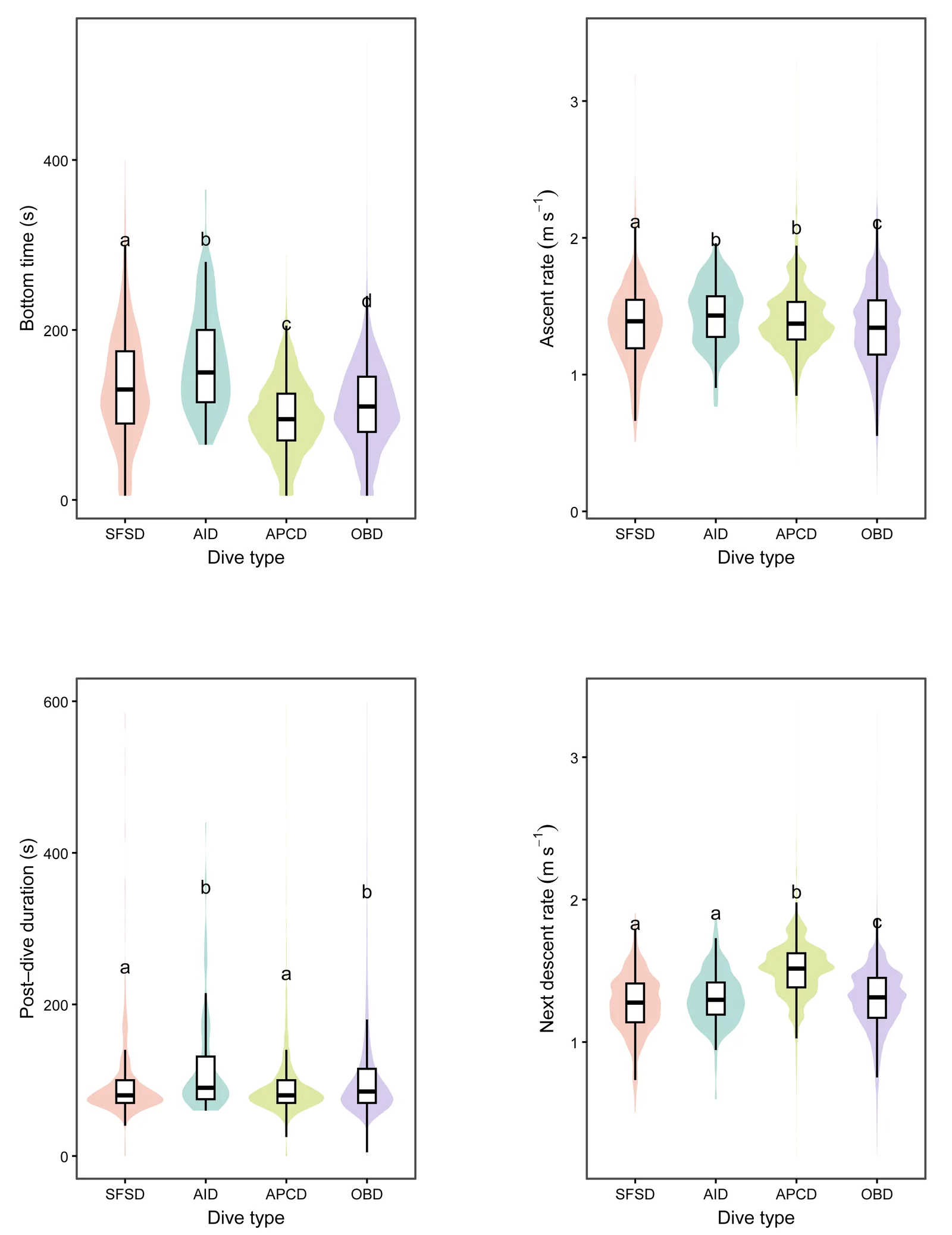
Distribution of dive cycle metrics by dive type for benthic dives in Australian fur seals (*Arctocephalus pusillus doriferus)*. Violin plots (colour-coded by dive type) show kernel density distributions with embedded boxplots for bottom time (top left), ascent rate (top right), post-dive duration (bottom left), and next descent rate (bottom right). Boxplots indicate the median (central line) and interquartile range (box); whiskers extend to 1.5 × IQR. Letters denote Tukey groupings within each metric (dive types sharing a letter are not significantly different at *P* < 0.05). Dive types: SFSD, Seafloor Stationary Dives; APCD, Attempted Prey Capture Dives; AID, Ambush Indicative Dives; OBD, Other Benthic Dives.

Estimated mean dive-cycle metrics differed among dive types, although the extent of separation varied among metrics (Table 2; Table S2). Relative to SFSD, AID dives had longer bottom times, whereas APCD and OBD had shorter bottom times. In relation to SFSD, AID and APCD exhibited faster ascent rates and did not clearly differ from one another, whereas OBD exhibited slightly slower ascent rates. In respect to SFSD, AID and OBD had longer post-dive durations and did not clearly differ from one another, whereas APCD did not differ from SFSD. Relative to SFSD, APCD exhibited a higher next descent rate, AID differed little from SFSD, and OBD exhibited a slightly lower next descent rate. Taken together, SFSD occupied an intermediate position for bottom time and vertical transit rates, while exhibiting comparatively short post-dive durations. This combination is consistent with a more compressed dive cycle (Table S2).

Quantification of run lengths for dive types (SFSD, AID, APCD) showed mostly single-dive SFSD, but multi-dive sequences also occurred (Fig. 3; Table S3). Maximal consecutive runs reached 29 for SFSD, 4 for AID, and 55 for APCD. Per individual permutation tests (10,000 randomizations preserving each individual’s dive type totals) identified clustering in a subset of AUFS for both SFSD and APCD. For SFSD, significant clustering was detected in four individuals, with observed mean run lengths consistently exceeding random expectation. For APCD, clustering was likewise evident in 10 individuals, whereas one additional individual showed a similar trend but was not significant after adjustment (Seal 8; adjusted *P* = 0.12). In contrast, AID were largely single events, with only one individual showing weak evidence of clustering.

Body size showed no evidence of influencing individual dive-type proportions when tested within the same GAM framework used for the temporal analyses. In binomial GAMs of hourly dive-type proportion among benthic dives, neither body mass nor SLEN was associated with SFSD proportion (mass: *β* = 0.077 ± 0.237 standard error of the mean (SE), *P* = 0.745; SLEN: *β* = −0.423 ± 0.359 SE, *P* = 0.238), and the complementary AID models yielded the same conclusion (mass: *β* = −0.077 ± 0.237 SE, *P* = 0.745; SLEN: *β* = 0.423 ± 0.359 SE, *P* = 0.238). These effects remained non-significant after Benjamini–Hochberg correction (adjusted *P* ≥ 0.477).

**Table 2:** Model-estimated means (±95% CI) for dive cycle metrics by dive type for benthic dives in Australian fur seals (*Arctocephalus pusillus doriferus*). Values are estimated marginal means from generalized additive models (GAMs) fitted separately for each metric with dive type as a fixed effect and individual identity included as a random intercept. Bottom time and post-dive duration were modeled using a Gamma distribution with a log link, ascent rate and next descent rate were modeled as log(1 + rate) with Gaussian errors for the transformed response log(1 + rate), and estimates and confidence intervals are back-transformed to m s^−^^1^ as exp(*η*) − 1. Durations are in seconds, ascent and next descent rates are in m s^−^^1^. “Next descent rate” refers to the vertical descent rate of the subsequent dive following the indexed dive. Dive types: AID, Ambush Indicative Dives; APCD, Attempted Prey Capture Dives; OBD, Other Benthic Dives; SFSD, Sea-floor Stationary Dives.

Dive type	Dive metric	Model family/link	Model-estimated mean (original units)	95% CI lower	95% CI upper
SFSD	Bottom time (s)	Gamma/log	152.549	141.398	164.580
AID	Bottom time (s)	Gamma/log	184.069	165.537	204.675
APCD	Bottom time (s)	Gamma/log	92.049	85.871	98.671
OBD	Bottom time (s)	Gamma/log	106.702	99.582	114.330
SFSD	Ascent rate (m s^−1^)	Gaussian on log(1 + rate)	1.353	1.289	1.419
AID	Ascent rate (m s^−1^)	Gaussian on log(1 + rate)	1.456	1.373	1.542
APCD	Ascent rate (m s^−1^)	Gaussian on log(1 + rate)	1.402	1.340	1.466
OBD	Ascent rate (m s^−1^)	Gaussian on log(1 + rate)	1.317	1.258	1.378
SFSD	Post-dive duration (s)	Gamma/log	89.981	77.256	104.803
AID	Post-dive duration (s)	Gamma/log	110.867	92.012	133.585
APCD	Post-dive duration (s)	Gamma/log	90.270	78.015	104.451
OBD	Post-dive duration (s)	Gamma/log	103.242	89.262	119.412
SFSD	Next descent rate (m s^−1^)	Gaussian on log(1 + rate)	1.305	1.263	1.348
AID	Next descent rate (m s^−1^)	Gaussian on log(1 + rate)	1.349	1.289	1.410
APCD	Next descent rate (m s^−1^)	Gaussian on log(1 + rate)	1.452	1.411	1.494
OBD	Next descent rate (m s^−1^)	Gaussian on log(1 + rate)	1.261	1.223	1.299

**Figure 3:**
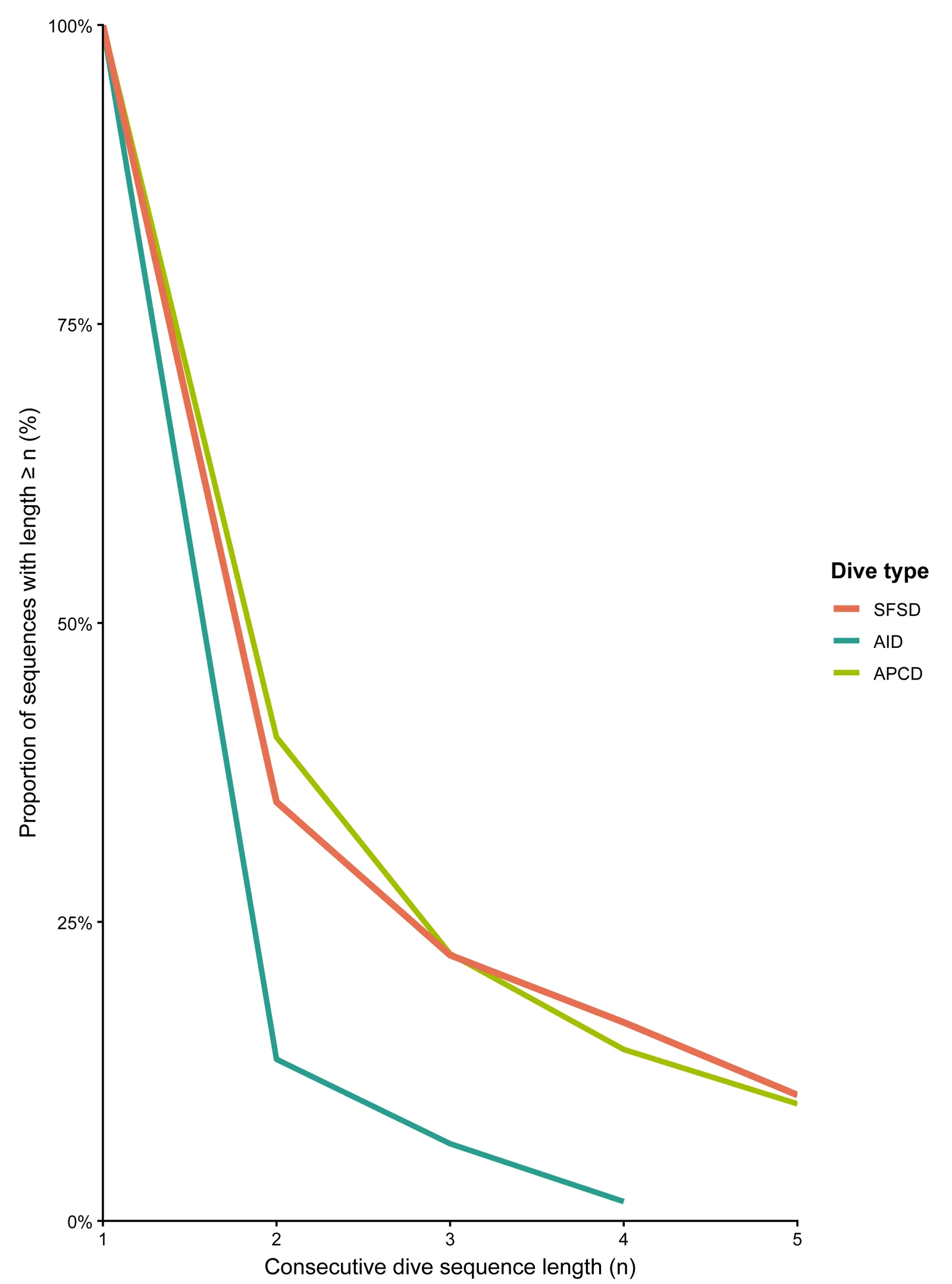
Consecutive sequence frequency by dive type in Australian fur seals (*Arctocephalus pusillus doriferus*). Line plot showing the proportion of sequences (runs) with length ≥*n* (*n* = 1–5) for Sea-floor Stationary Dives (SFSD, orange), Attempted Prey Capture Dives (APCD, green), and Ambush Indicative Dives (AID, teal). The *y*-axis gives the proportion of sequences with length ≥ *n*, and the *x*-axis gives consecutive sequence length.

The frequency of SFSD displayed clear temporal variation (Fig. 4). The cyclic smooth for hour was significant (effective degrees of freedom (edf) = 3.91, *χ*^2^ = 73.92, *P* = 1.42 × 10^−6^), consistent with a pronounced bimodal pattern with peaks near dawn and dusk (*R*^2^adj = 0.44; deviance explained = 61.7%; *n* = 697). In contrast, the rare AID displayed no detectable temporal pattern (edf = 4.70, *P* = 0.546; *R*^2^adj = 0.22; deviance explained = 31.1%; *n* = 104). In both models, the random effect for individual identity was highly significant (SFSD: *χ*^2^ = 14.16, *P* = 0.0017; AID: *χ*^2^ = 99.4, *P* = 0.000000087), indicating substantial intra-individual heterogeneity in frequency and timing.

**Figure 4:**
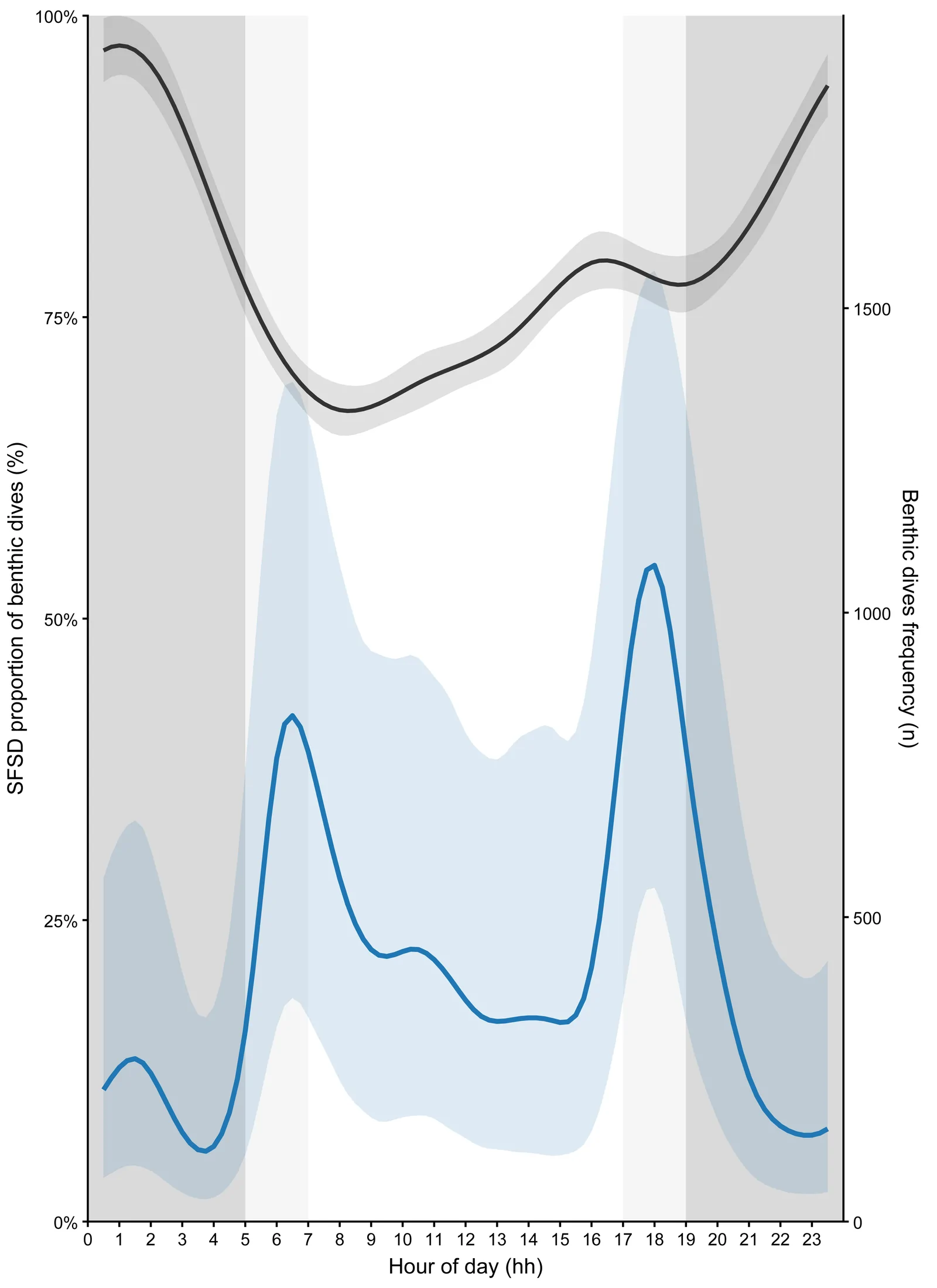
Temporal variation in SFSD proportion predicted by a cyclic GAM with individual random effects and daytime benthic dive frequency in Australian fur seals. The blue curve shows the estimated smooth for hour of day (0–23 h) on the response scale with 95% confidence intervals (shaded). The black curve shows the estimated smooth for hour of day for benthic dive frequency with 95% confidence intervals (shaded). Night-time (dark grey) and dawn/dusk (light grey) periods are indicated.

Visual inspection of the SFSD locations in the two females instrumented with a GPS data logger revealed spatial clustering supporting the findings of the sequence analysis. This was observed across multiple trips (Fig. 5).

**Figure 5:**
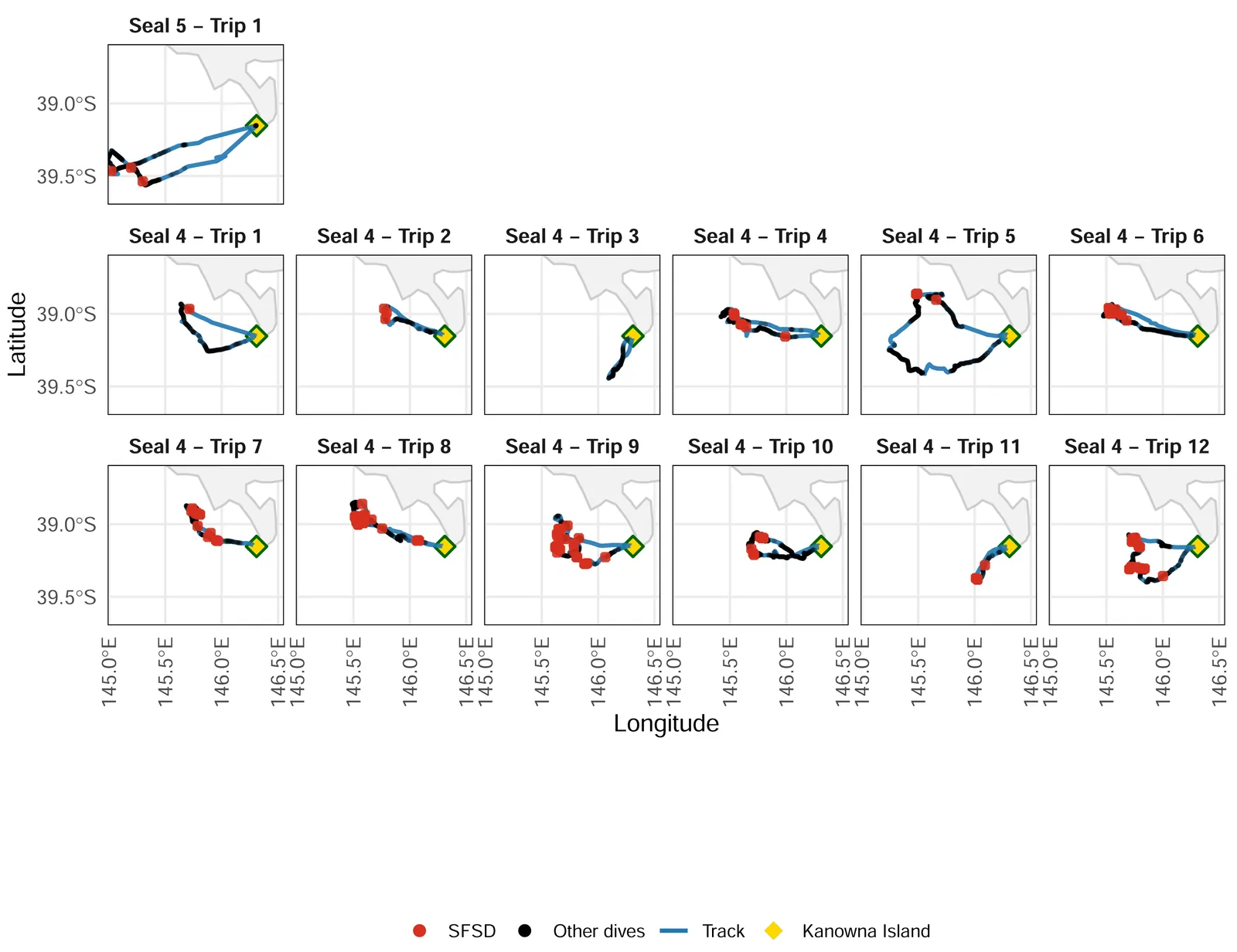
GPS tracks for Seal 4 and 5, showing mapped dive locations by classification in Australian fur seals (*Arctocephalus pusillus doriferus*). The blue line denotes the at-sea track reconstructed from sequential GPS fixes, red circles mark locations where Sea-floor Stationary Dives (SFSD) occurred, black points indicate other benthic dives, the yellow diamond represents Kanowna Island (39°10′S, 146°18′E). Source data and map background: Natural Earth (https://www.naturalearthdata.com/).

## Discussion

For air-breathing benthic divers, remaining motionless on the seafloor is likely to be costly because oxygen budgets are limited and time spent stationary is not available for searching or handling prey (Carbone & Houston, 1996; Heithaus & Frid, 2003). The preliminary video observation of AUFS individuals engaging in stationary behavior on the seafloor during foraging trips was, therefore, of interest. In the present study, swim-speed/dive data revealed a low but widespread occurrence of stationary seafloor behavior during benthic dives in all adult female AUFS studied. While a small proportion of these dives appear consistent with ambush predation attempts, the majority did not exhibit the dive characteristics typically associated with rest or recovery. Instead, most stationary seafloor dives showed distinct dive-cycle features and temporal clustering, suggesting that they may represent another context-dependent behavioral response during benthic foraging, potentially linked to predator-risk mitigation.

### Prevalence and function of stationary seafloor behavior

Dives in which stationary seafloor periods were immediately followed by rapid movement (AID) had the longest bottom phases and post-dive durations. This is consistent with stationary ambush predation attempts and extended recovery (Platell & Potter, 1998; Wearmouth et al., 2014). Such dives were observed in less than half of the study animals and, within these, there was considerable inter-individual variation. This could reflect varying levels of individual foraging specialization in ambush predation (Matich, Heithaus & Layman, 2011). In addition, within AUFS displaying such dives, there was considerable intra-individual variation in their prevalence between foraging trips. This could reflect variability in visitation of habitats associated with the potential for ambush predation (Bartes et al., 2025; Scharf, 2024). However, in general, the proportion of dives with stationary seafloor periods that were classified as apparent ambush predation events was low, suggesting other drivers for this behavior.

In many diving mammals, resting or recovery dives are typically characterized by slow, passive descents, and extended post-dive durations (Boyd, 1997; Doniol-Valcroze et al., 2011). These features allow for physiological recovery following periods of high activity or oxygen depletion (Ramasco, Biuw & Nilssen, 2014; Kendall-Bar, Williams & Costa, 2023). In contrast, SFSD in the present study showed rapid descent–ascent phases, intermediate bottom durations relative to the other dive types, and no evidence of prolonged post-dive recovery. While brief immobility on the seafloor could provide minor energetic relief, the absence of extended surface recovery suggests that SFSD did not primarily function as rest or recovery dives (Christiansen et al., 2015).

Predation risk is generally spatially and temporally structured, and prey are expected to trade foraging efficiency for safety when predators are detected or anticipated (Ferrari, Sih & Chivers, 2009). More broadly, marine meso-predators may balance energetic demands against predation risk across changing environmental conditions, producing state-dependent behavioral adjustments that reflect this trade-off (Beltran et al., 2021; Hale et al., 2026). For pinnipeds, the surface and upper water column are especially dangerous because predators can approach from multiple directions and prey are silhouetted against downwelling light (Heithaus & Frid, 2003). The dive metrics associated with the non-ambush stationary seafloor behavior in the present study, with seafloor durations longer than in foraging dives followed by very short surface intervals, are consistent with a risk-mitigation response to elevated predation risk.

At broader scales, similar risk-mitigation responses can occur once predation risk is perceived, with individuals moving inshore or into structurally complex habitats to avoid predator overlap and constrain approach, thereby reducing encounter rates and improving detectability under low-visibility conditions (Sironi et al., 2008; Srinivasan, 2019). These shifts exploit predictable features, such as background complexity and restricted approach trajectories, that reduce ambush effectiveness (Fouts & Nelson, 1999; Říha et al., 2021; South et al., 2017). Comparable predator-driven responses have been documented in other marine vertebrates, including humpback whales (*Megaptera novaeangliae*) moving closer to shore following killer whale (*Orcinus orca*) sightings (Pitman et al., 2015) and beaked whales showing marked changes in diving coordination and predator-avoidance trade-offs under killer whale risk (Aguilar de Soto et al., 2020; Siegal et al., 2022). Similarly, the stationary seafloor behavior observed in AUFS in the present study may reflect a short-term reaction to elevated risk with the same functional outcome. Because attacks by known AUFS predators, such as white sharks (*Carcharodon carcharias*) (Kirkwood & Dickie, 2005), are usually initiated from below and to the rear near the surface (Dunphy-Daly et al., 2010; Heithaus & Frid, 2003; Heithaus et al., 2007), staying on the seafloor could reduce surface exposure and narrow the range of possible attack angles.

### Temporal and spatial distribution of stationary seafloor behavior

No association was detected between body size and the frequency of SFSD or AID, suggesting that variation in stationary seafloor behavior among the adult females sampled was not strongly structured by body mass or standard length. This may indicate that the occurrence of this behavior depends more on local context during foraging trips than on individual morphology (Arnould et al., 2015; Bartes et al., 2025; Hale et al., 2026).

Clustering of anti-predator behavior reflects state dependence: once risk is detected, individuals maintain heightened caution until cues decay or the predator leaves (De Vos et al., 2015a; De Vos et al., 2015b; Loflen & Hovel, 2010). Such persistence reallocates time from feeding to risk management, with potential consequences for intake and energetic balance within the same trip (Matassa et al., 2016; Sirot, 2019). In the present study, while individuals were observed to adopt stationary seafloor behavior in mostly isolated events, such dives commonly occurred in temporally clustered sequential dives (Hale et al., 2026). This suggests that, once initiated, stationary seafloor behavior may persist across short sequences of dives, consistent with a short-term response to elevated risk. While SFSD comprised only a small proportion of dives and overall trip duration, even brief interruptions can reduce effective search time and lower intake in central-place foragers, particularly when prey are patchily distributed and foraging is constrained by commuting distance and oxygen budgets (Orians, 1979). More broadly, risk effects are known to generate missed-opportunity costs and alter patch use (Beltran et al., 2021; Eccard & Liesenjohann, 2014).

The stationary seafloor behavior observed in individuals in the present study displayed a strong crepuscular distribution. In the marine environment, twilight periods are marked by rapid shifts in ambient light, increased surface glare, and changing contrast gradients that alter visual detection ranges in the water column (Green, Akins & Côté, 2011; Meager et al., 2010; Regular, Hedd & Montevecchi, 2011). These optical transitions typically reduce prey reaction distances while still permitting ambush predators with low-light adaptations to close distance effectively (Campanella et al., 2019; Rickel & Genin, 2005). Indeed, white sharks show crepuscular activity peaks and exploit light-mediated advantages by hunting at sunrise and sunset when prey silhouettes are enhanced and detection asymmetries favor upward or oblique attack (Jewell et al., 2024; Watanabe et al., 2019). In predator–prey systems, such temporal windows often represent predictable spikes in encounter probability, and prey are expected to adjust behavior when predators are present (Ferrari, Sih & Chivers, 2009; Hale et al., 2026; Higginson et al., 2012). Therefore, the increase in SFSD observed at dawn and dusk in the present study is consistent with stationary seafloor behavior occurring during periods of elevated risk, including the possibility of increased encounters with predatory sharks at these times.

While data on the spatial distribution of stationary seafloor behavior was available for only two individuals in the present study, they provide insightful information. The numerous trips to the same general region by Seal 4 are consistent with benthic foraging pinnipeds displaying a degree of individual foraging specialization and habitat-use fidelity (Baylis et al., 2015; Chilvers, 2008). Interestingly, SFSD were repeatedly recorded for this individual in the same small portion of the habitat used. While this suggests that the conditions associated with SFSD may have been localized, it also suggests repeated use of an area despite the occurrence of SFSD, potentially reflecting a trade-off between local habitat value and avoidance behavior (Balaban-Feld et al., 2019; Steinhoff et al., 2020). Such decisions within “seascapes of fear” (Frid, Marliave & Heithaus, 2012; Wirsing, Heithaus & Dill, 2011; Wirsing et al., 2008) can influence benthic habitat-use, local prey consumption patterns, prey refuge-use and the distribution of foraging effort throughout the habitat of the species (Dunphy-Daly et al., 2010; Groot, 2022; Heithaus et al., 2009).

Interestingly, while only one SFSD was recorded for Seal 5, this event occurred in the same general area as those observed in Seal 4. Recent studies suggest white shark movements within Bass Strait are region-specific, which could lead to increased encounter rates by AUFS in some areas (Burke et al., 2025). Indeed, white sharks are known to sequentially visit seal colonies (De Vos et al., 2015a; De Vos et al., 2015b; Wcisel et al., 2015) and the area where SFSD were recorded in the present study lies in the ∼126 km between the Seal Rocks (38°31′S, 145°05′E) and Kanowna Island colonies. In addition, the SFSD observed in AUFS could result from the presence of other predatory sharks, such as the broadnose sevengill shark (*Notorynchus cepedianus*) where large individuals are known to consume fur seals and strong site fidelity can generate restricted “risk hotspots” (Barnett et al., 2010; Braccini, 2008). However, additional information on the spatial distribution of stationary seafloor behavior, and concurrent movement patterns of predatory sharks in the region, are needed to better understand how these may influence AUFS movement corridors, patch use, and habitat preference.

## Conclusions

The results of the present study indicate that stationary seafloor behavior is a distinct but infrequent component of benthic diving in adult female AUFS. A small proportion of these dives were consistent with ambush predation attempts, whereas most showed dive-cycle characteristics inconsistent with rest or recovery and were associated with temporal clustering, a crepuscular pattern, and localized spatial recurrence. Together, these findings suggest that stationary seafloor behavior represents a context-dependent response during benthic foraging, with predator-risk mitigation as a plausible explanation. This behavior reflects a trade-off between foraging and safety under physiological constraints in an air-breathing benthic diver. Correspondingly, such behavior could also be present in other pinniped meso-predators (Carter et al., 2016; Perrin, Würsig & Thewissen, 2009), with potential impacts on fine-scale habitat use. Additional studies combining GPS tracking and swim speed/dive data logging are needed to further determine the spatial distribution of this behavior in AUFS and whether it is associated with specific seafloor habitats and/or geographic regions. Similarly, more information on the fine-scale temporal and spatial distribution of potential AUFS predators, in conjunction with environmental covariates (*e.g.*, season, light regime, turbidity, seafloor complexity), is needed to better understand encounter-driven risk responses in the species. In particular, future studies should investigate the sensory or environmental cues that initiate stationary seafloor behavior, including whether responses are triggered by direct predator detection, indirect cues, or changing visual conditions. Incorporating such knowledge on predator risk states is required for improving the predictions of bioenergetic and movement models in the rapidly-changing ecosystem of Bass Strait (Speakman, McHuron & Arnould, 2024; Walker et al., 2021).

## Supplemental Information

10.7717/peerj.21782/supp-1 Video S1 Example of apparent ambush predation by a female Australian fur seal (*Arctocephalus pusillus doriferus*) The seal remains stationary near the seafloor for a brief period of time before initiating a rapid chase after detecting prey and successfully capturing it.

10.7717/peerj.21782/supp-2 Video S2 Example of a stationary seafloor phase without an obvious prey pursuit by a female Australian fur seal (*Arctocephalus pusillus doriferus*) The seal remains completely still, lying flat on the seafloor throughout the dive, while repeatedly looking upward into the water column.

10.7717/peerj.21782/supp-3 Table S1 Trip-level summary of benthic dive activity and classifications for adult female Australian fur seals (*Arctocephalus pusillus doriferus*) Columns report AUFS ID (Seal), trip identifier (sequential per individual), trip duration (h), total benthic dives (*n*), counts of Sea-floor Stationary Dives (SFSD, *n*), per-trip proportions of benthic dives classified as SFSD (%), per-trip proportions of trip duration performing SFSD (%), count of Ambush Indicative Dives (AID, *n*), and per-trip proportions of benthic dives classified as AID (%). SFSD were defined as dives with ≥50% of the bottom phase at zero swim speed. AID were identified from swim-speed signatures indicative of rapid strike behavior (≥1.0 ms^−1^). Dive-type proportions (% benthic dives) were calculated as (count ÷ total benthic dives) × 100. SFSD proportion trip duration (%) was calculated as (time in SFSD ÷ trip duration) ×100.

10.7717/peerj.21782/supp-4 Table S2 Tukey-adjusted pairwise comparisons of dive cycle metrics across dive types from GAMs in Australian fur seals (*Arctocephalus pusillus doriferus)* For each metric, rows report Tukey-adjusted pairwise contrasts between dive types derived from generalized additive models (GAMs) fitted separately for each response with dive type as a fixed effect and individual identity included as a random intercept. For bottom time and post-dive duration (Gamma models with a log link), contrasts are reported as response-scale ratios (Dive type 1 ÷ Dive type 2), with percent change calculated as (ratio − 1) × 100. For ascent rate and next descent rate, models were fitted using Gaussian errors for the transformed response log(1 +rate), contrasts are reported as differences on the *η* = log(1 +rate) scale (Dive type 1 − Dive type 2), with percent change in (1 +rate) calculated as (exp(estimate) − 1) × 100. Standard errors and degrees of freedom are from the estimated marginal means framework. *P*-values are Tukey-adjusted within each metric. Durations are in seconds, ascent and next descent rates are in m s^−1^. “Next descent rate” refers to the vertical descent rate of the subsequent dive following the indexed dive. Dive types: AID, Ambush Indicative Dives; APCD, Attempted Prey Capture Dives; OBD, Other Benthic Dives; SFSD, Sea-floor Stationary Dives.

10.7717/peerj.21782/supp-5 Table S3 Run-length clustering statistics for benthic dive types by individual Australian fur seals (*Arctocephalus pusillus doriferus*) For each AUFS ID and dive type (SFSD, APCD, AID), contiguous sequences (“runs”) were identified from the chronological dive record. Columns report number of runs (*n*), median and maximum run length, observed mean run length, permutation mean run length from 10,000 randomizations preserving each individual’s dive-type totals, empirical one-sided *p*-value (probability that permuted mean ≥ observed mean), effect ratio (observed ÷ permuted mean; >1 indicates clustering), and Benjamini–Hochberg adjusted *p*-value within each dive type. Blank entries indicate insufficient events to compute runs (≤1 occurrence). *P*-values reported as 0 indicate *p* < 1 × 10^−4^ at the permutation resolution.

## Additional Information and Declarations

### Data Availability

The following information was supplied regarding data availability: The data is available at Zenodo: - Arnould J, & Salzeri P. (2026). The hunter or the hunted? Inferring benthic meso-predator behavior from swim velocity data (source data) [Data set]. Zenodo. https://doi.org/10.5281/zenodo.18973602.
