# Supplementary material for: The hunter or the hunted? Inferring benthic meso-predator behavior from swim velocity data
Source: PeerJ. 2026 Oct 6;14:e21782. doi: 10.7717/peerj.21782 (PMC13651780; doi:10.7717/peerj.21782)
Supplement: Supplementary file 1 [file peerj-14-21782-s003.docx]

**Table S3. Trip level summary of benthic dive activity and classifications for adult female Australian fur seals (Arctocephalus pusillus doriferus).**

Columns report AUFS ID (Seal), trip identifier (sequential per individual), trip duration (h), total benthic dives (n), counts of Sea floor Stationary Dives (SFSD, n), per trip proportions of benthic dives classified as SFSD (%), per trip proportions of trip duration performing SFSD (%), count of Ambush Indicative Dives (AID, n), and per trip proportions of benthic dives classified as AID (%). SFSD were defined as dives with ≥50% of the bottom phase at zero swim speed. AID were identified from swim speed signatures indicative of rapid strike behavior (≥1.0 m s⁻¹). Dive-type proportions (% benthic dives) were calculated as (count ÷ total benthic dives) × 100. SFSD proportion trip duration (%) was calculated as (time in SFSD ÷ trip duration) × 100.

| Seal | Trip | Trip duration  (h) | Benthic dives (n) | SFSD (n) | SFSD  (% benthic dives) | SFSD proportion trip duration (%) | AID (n) | AID  (% benthic dives) |
| --- | --- | --- | --- | --- | --- | --- | --- | --- |
| 1 | 1 | 63.91 | 506 | 1 | 0.20 | 0.07 | 0 | 0 |
|  | 2 | 33.15 | 342 | 4 | 1.17 | 4.88 | 2 | 0.58 |
|  | 3 | 57.60 | 513 | 0 | 0 | 0 | 0 | 0 |
|  | 4 | 84.85 | 698 | 16 | 2.29 | 2.03 | 3 | 0.43 |
|  | 5 | 196.18 | 1281 | 15 | 1.17 | 0.58 | 0 | 0 |
|  | 6 | 155.76 | 1144 | 9 | 0.79 | 1.83 | 0 | 0 |
|  | 7 | 115.67 | 794 | 38 | 4.79 | 12.57 | 0 | 0 |
|  | 8 | 131.01 | 982 | 4 | 0.41 | 0.32 | 0 | 0 |
|  | 9 | 106.49 | 743 | 16 | 2.15 | 12.74 | 5 | 0.67 |
|  | 10 | 82.85 | 658 | 0 | 0 | 0 | 0 | 0 |
| 2 | 1 | 102.23 | 577 | 0 | 0 | 0 | 0 | 0 |
|  | 2 | 136.95 | 996 | 0 | 0 | 0 | 0 | 0 |
|  | 3 | 176.36 | 1092 | 0 | 0 | 0 | 0 | 0 |
|  | 4 | 109.43 | 650 | 0 | 0 | 0 | 0 | 0 |
|  | 5 | 152.60 | 990 | 0 | 0 | 0 | 0 | 0 |
|  | 6 | 209.46 | 767 | 2 | 0.26 | 0.09 | 0 | 0 |
|  | 7 | 86.59 | 543 | 4 | 0.74 | 0.49 | 1 | 0.18 |
|  | 8 | 210.01 | 1258 | 0 | 0 | 0 | 0 | 0 |
| 3 | 1 | 102.52 | 548 | 3 | 0.55 | 0.83 | 0 | 0 |
| 4 | 1 | 102.18 | 153 | 1 | 0.65 | 0.08 | 0 | 0 |
|  | 2 | 82.03 | 180 | 3 | 1.67 | 0.28 | 0 | 0 |
|  | 3 | 94.49 | 431 | 15 | 3.48 | 2.19 | 3 | 0.70 |
|  | 4 | 82.28 | 417 | 6 | 1.44 | 0.61 | 0 | 0 |
|  | 5 | 95.65 | 625 | 49 | 7.84 | 6.81 | 6 | 0.96 |
|  | 6 | 77.93 | 568 | 25 | 4.40 | 5.18 | 2 | 0.35 |
|  | 7 | 73.37 | 492 | 117 | 23.78 | 21.13 | 12 | 2.44 |
|  | 8 | 64.25 | 493 | 44 | 8.92 | 17.84 | 4 | 0.81 |
|  | 9 | 59.62 | 493 | 15 | 3.04 | 4.22 | 0 | 0 |
|  | 10 | 120.40 | 603 | 27 | 4.48 | 20.87 | 1 | 0.17 |
|  | 11 | 98.10 | 533 | 70 | 13.13 | 7.26 | 17 | 3.19 |
|  | 12 | 92.20 | 763 | 110 | 14.42 | 12.88 | 31 | 4.06 |
| 5 | 1 | 146.52 | 341 | 3 | 0.88 | 0.88 | 0 | 0 |
| 6 | 1 | 110.69 | 770 | 3 | 0.39 | 10.01 | 0 | 0 |
| 7 | 1 | 120.22 | 324 | 1 | 0.31 | 0.09 | 0 | 0 |
|  | 2 | 38.54 | 264 | 11 | 4.17 | 3.33 | 0 | 0 |
|  | 3 | 37.69 | 223 | 0 | 0 | 0 | 0 | 0 |
| 8 | 1 | 57.12 | 340 | 0 | 0 | 0 | 0 | 0 |
|  | 2 | 120.30 | 1011 | 17 | 1.68 | 3.25 | 3 | 0.30 |
| 9 | 1 | 82.94 | 601 | 2 | 0.33 | 0.17 | 0 | 0 |
|  | 2 | 50.19 | 373 | 16 | 4.29 | 26.42 | 0 | 0 |
| 10 | 1 | 76.30 | 381 | 2 | 0.52 | 0.22 | 0 | 0 |
| 11 | 1 | 201.66 | 581 | 10 | 1.72 | 0.63 | 2 | 0.34 |
|  | 2 | 145.58 | 662 | 5 | 0.76 | 2.75 | 0 | 0 |
|  | 3 | 167.17 | 838 | 5 | 0.60 | 6.01 | 4 | 0.48 |
|  | 4 | 182.38 | 891 | 4 | 0.45 | 0.29 | 0 | 0 |
|  | 5 | 27.27 | 200 | 4 | 2.00 | 2.26 | 0 | 0 |
|  | 6 | 207.82 | 1214 | 8 | 0.66 | 0.70 | 7 | 0.58 |
|  | 7 | 61.14 | 358 | 1 | 0.28 | 0.30 | 0 | 0 |
|  | 8 | 70.22 | 286 | 1 | 0.35 | 1.03 | 0 | 0 |
|  | 9 | 80.52 | 280 | 1 | 0.36 | 0.16 | 0 | 0 |
| 12 | 1 | 37.56 | 198 | 0 | 0 | 0 | 0 | 0 |
|  | 2 | 44.03 | 283 | 0 | 0 | 0 | 0 | 0 |
|  | 3 | 217.18 | 1510 | 6 | 0.40 | 0.20 | 0 | 0 |
|  | 4 | 73.33 | 611 | 0 | 0 | 0 | 0 | 0 |
|  | 5 | 103.21 | 789 | 1 | 0.13 | 0.06 | 0 | 0 |
|  | 6 | 175.07 | 1053 | 1 | 0.09 | 0.08 | 0 | 0 |
|  | 7 | 135.68 | 854 | 0 | 0 | 0 | 0 | 0 |
|  | 8 | 106.01 | 713 | 1 | 0.14 | 0.09 | 1 | 0.14 |
|  | 9 | 156.08 | 1020 | 0 | 0 | 0 | 0 | 0 |
