# Supplementary material for: The hunter or the hunted? Inferring benthic meso-predator behavior from swim velocity data
Source: PeerJ. 2026 Oct 6;14:e21782. doi: 10.7717/peerj.21782 (PMC13651780; doi:10.7717/peerj.21782)
Supplement: Supplementary file 2 [file peerj-14-21782-s004.docx]

**Table S4. Tukey-adjusted pairwise comparisons of dive cycle metrics across dive types from GAMs in Australian fur seals (*Arctocephalus pusillus doriferus)*.**

For each metric, rows report Tukey-adjusted pairwise contrasts between dive types derived from generalized additive models (GAMs) fitted separately for each response with dive type as a fixed effect and individual identity included as a random intercept. For bottom time and post-dive duration (Gamma models with a log link), contrasts are reported as response-scale ratios (Dive type 1 ÷ Dive type 2), with percent change calculated as (ratio − 1) × 100. For ascent rate and next descent rate, models were fitted using Gaussian errors for the transformed response log(1 + rate), contrasts are reported as differences on the η = log(1 + rate) scale (Dive type 1 − Dive type 2), with percent change in (1 + rate) calculated as (exp(estimate) − 1) × 100. Standard errors and degrees of freedom are from the estimated marginal means framework. P-values are Tukey-adjusted within each metric. Durations are in seconds, ascent and next descent rates are in m s⁻¹. “Next descent rate” refers to the vertical descent rate of the subsequent dive following the indexed dive. Dive types: AID = Ambush Indicative Dives; APCD = Attempted Prey Capture Dives; OBD = Other Benthic Dives; SFSD = Sea-floor Stationary Dives.

| Dive metric | Contrast (dive1 vs dive2) | Scale | Estimate | Percent change | Standard error | Degrees of freedom | Tukey-adjusted p |
| --- | --- | --- | --- | --- | --- | --- | --- |
| Bottom time (s) | SFSD / AID | Ratio (type1/type2) | 0.8287615 | -17.12% | 0.036413848 | 37692.13 | 1.13*10^-4^ |
| Bottom time (s) | SFSD / APCD | Ratio (type1/type2) | 1.6572618 | +65.73% | 0.027908965 | 37692.13 | < 1*10^-16^ |
| Bottom time (s) | SFSD / OBD | Ratio (type1/type2) | 1.4296788 | +42.97% | 0.023453257 | 37692.13 | < 1*10^-16^ |
| Bottom time (s) | AID / APCD | Ratio (type1/type2) | 1.9996849 | +99.97% | 0.082766643 | 37692.13 | < 1*10^-16^ |
| Bottom time (s) | AID / OBD | Ratio (type1/type2) | 1.7250787 | +72.51% | 0.071092738 | 37692.13 | < 1*10^-16^ |
| Bottom time (s) | APCD / OBD | Ratio (type1/type2) | 0.8626753 | -13.73% | 0.004391325 | 37692.13 | < 1*10^-16^ |
| Ascent rate (m s^-1^) | SFSD − AID | Diff on eta=log(1+rate) | -0.04304142 | -4.21% in (1+rate) | 0.012212739 | 37770.07 | 2.40*10^-3^ |
| Ascent rate (m s^-1^) | SFSD − APCD | Diff on eta=log(1+rate) | -0.02071766 | -2.05% in (1+rate) | 0.004676364 | 37770.07 | 5.56*10^-5^ |
| Ascent rate (m s^-1^) | SFSD − OBD | Diff on eta=log(1+rate) | 0.01526969 | +1.54% in (1+rate) | 0.004556728 | 37770.07 | 4.47*10^-3^ |
| Ascent rate (m s^-1^) | AID − APCD | Diff on eta=log(1+rate) | 0.02232376 | +2.26% in (1+rate) | 0.011504916 | 37770.07 | 2.11*10^-1^ |
| Ascent rate (m s^-1^) | AID − OBD | Diff on eta=log(1+rate) | 0.05831110 | +6.00% in (1+rate) | 0.011456000 | 37770.07 | 2.14*10^-6^ |
| Ascent rate (m s^-1^) | APCD − OBD | Diff on eta=log(1+rate) | 0.03598735 | +3.66% in (1+rate) | 0.001409634 | 37770.07 | < 1*10^-16^ |
| Post-dive duration (s) | SFSD / AID | Ratio (type1/type2) | 0.8116162 | -18.838% | 0.051568950 | 37729.06 | 5.63*10^-3^ |
| Post-dive duration (s) | SFSD / APCD | Ratio (type1/type2) | 0.9967973 | -0.32% | 0.024270135 | 37729.06 | 9.99*10^-1^ |
| Post-dive duration (s) | SFSD / OBD | Ratio (type1/type2) | 0.8715579 | -12.84% | 0.020676422 | 37729.06 | 4.10*10^-8^ |
| Post-dive duration (s) | AID / APCD | Ratio (type1/type2) | 1.2281634 | +22.82% | 0.073507855 | 37729.06 | 3.33*10^-3^ |
| Post-dive duration (s) | AID / OBD | Ratio (type1/type2) | 1.0738547 | +7.39% | 0.063997813 | 37729.06 | 6.30*10^-1^ |
| Post-dive duration (s) | APCD / OBD | Ratio (type1/type2) | 0.8743582 | -12.56% | 0.006419691 | 37729.06 | < 1*10^-16^ |
| Next descent rate (m s^-1^) | SFSD − AID | Diff on eta=log(1+rate) | -0.01865417 | -1.85% in (1+rate) | 0.010486902 | 37775.13 | 2.84*10^-1^ |
| Next descent rate (m s^-1^) | SFSD − APCD | Diff on eta=log(1+rate) | -0.06175900 | -5.99% in (1+rate) | 0.004015707 | 37775.13 | < 1*10^-16^ |
| Next descent rate (m s^-1^) | SFSD − OBD | Diff on eta=log(1+rate) | 0.01940617 | +1.96% in (1+rate) | 0.003913045 | 37775.13 | 4.22*10^-6^ |
| Next descent rate (m s^-1^) | AID − APCD | Diff on eta=log(1+rate) | -0.04310484 | -4.22% in (1+rate) | 0.009879032 | 37775.13 | 7.56*10^-5^ |
| Next descent rate (m s^-1^) | AID − OBD | Diff on eta=log(1+rate) | 0.03806033 | +3.88% in (1+rate) | 0.009837027 | 37775.13 | 6.32*10^-4^ |
| Next descent rate (m s^-1^) | APCD − OBD | Diff on eta=log(1+rate) | 0.08116517 | +8.46% in (1+rate) | 0.001210268 | 37775.13 | < 1*10^-16^ |
