# Supplementary material for: The hunter or the hunted? Inferring benthic meso-predator behavior from swim velocity data
Source: PeerJ. 2026 Oct 6;14:e21782. doi: 10.7717/peerj.21782 (PMC13651780; doi:10.7717/peerj.21782)
Supplement: Supplementary file 3 [file peerj-14-21782-s005.docx]

**Table S5. Run length clustering statistics for benthic dive types by individual Australian fur seals (Arctocephalus pusillus doriferus).**

For each AUFS ID and dive type (SFSD, APCD, AID), contiguous sequences (“runs”) were identified from the chronological dive record. Columns report number of runs (n), median and maximum run length, observed mean run length, permutation mean run length from 10,000 randomizations preserving each individual’s dive type totals, empirical one sided p value (probability that permuted mean ≥ observed mean), effect ratio (observed ÷ permuted mean; >1 indicates clustering), and Benjamini–Hochberg adjusted p value within each dive type. Blank entries indicate insufficient events to compute runs (≤1 occurrence). P values reported as 0 indicate p < 1×10⁻⁴ at the permutation resolution.

| Seal | Dive type | Number of runs (n) | Median run length | Maximum run length | Observed mean | Permutation mean | Empirical p-value | Effect ratio | Adjusted p-value |
| --- | --- | --- | --- | --- | --- | --- | --- | --- | --- |
| 1 | AID | 10 | 1 | 1 | 1 | 1.058654 | 1 | 0.944596 | 1 |
|  | APCD | 1096 | 1 | 12 | 1.677007 | 1.442297 | 0 | 1.162734 | 0 |
|  | SFSD | 31 | 1 | 29 | 3.322581 | 1.150691 | 0 | 2.887464 | 0 |
| 2 | AID | 1 | 1 | 1 | 1 | 1 | 1 | 1 | 1 |
|  | APCD | 793 | 1 | 32 | 2.617907 | 1.845924 | 0 | 1.418209 | 0 |
|  | SFSD | 6 | 1 | 1 | 1 | 1.00244 | 1 | 0.997566 | 1 |
| 3 | AID |  |  |  |  |  |  |  |  |
|  | APCD | 83 | 1 | 16 | 2.072289 | 1.702758 | 1.00E-04 | 1.217019 | 0.00025 |
|  | SFSD | 3 | 1 | 1 | 1 | 1 | 1 | 1 | 1 |
| 4 | AID | 62 | 1 | 4 | 1.225806 | 1.117436 | 0.0128 | 1.096982 | 0.0256 |
|  | APCD | 583 | 1 | 17 | 1.998285 | 1.598969 | 0 | 1.249734 | 0 |
|  | SFSD | 216 | 1 | 28 | 2.231481 | 1.579257 | 0 | 1.412994 | 0 |
| 5 | AID |  |  |  |  |  |  |  |  |
|  | APCD | 14 | 1 | 2 | 1.071429 | 1.020913 | 0.2824 | 1.049481 | 0.445895 |
|  | SFSD | 3 | 1 | 1 | 1 | 1 | 1 | 1 | 1 |
| 6 | AID |  |  |  |  |  |  |  |  |
|  | APCD | 39 | 1 | 4 | 1.333333 | 1.13158 | 0.0025 | 1.178294 | 0.005769 |
|  | SFSD | 3 | 1 | 1 | 1 | 1 | 1 | 1 | 1 |
| 7 | AID |  |  |  |  |  |  |  |  |
|  | APCD | 58 | 1 | 5 | 1.310345 | 1.119122 | 1.00E-04 | 1.170869 | 0.00025 |
|  | SFSD | 6 | 1.5 | 4 | 2 | 1.311003 | 0.0078 | 1.52555 | 0.016714 |
| 8 | AID | 3 | 1 | 1 | 1 | 1 | 1 | 1 | 1 |
|  | APCD | 86 | 1 | 4 | 1.313953 | 1.237925 | 0.064 | 1.061416 | 0.12 |
|  | SFSD | 12 | 1 | 3 | 1.416667 | 1.039474 | 1.00E-04 | 1.362869 | 0.00025 |
| 9 | AID |  |  |  |  |  |  |  |  |
|  | APCD | 109 | 1 | 27 | 2.522936 | 2.023185 | 0 | 1.247012 | 0 |
|  | SFSD | 17 | 1 | 2 | 1.058824 | 1.030934 | 0.4201 | 1.027053 | 0.63015 |
| 10 | AID |  |  |  |  |  |  |  |  |
|  | APCD | 51 | 1 | 11 | 2.137255 | 1.553112 | 0 | 1.376111 | 0 |
|  | SFSD | 2 | 1 | 1 | 1 | 1 | 1 | 1 | 1 |
| 11 | AID | 12 | 1 | 2 | 1.083333 | 1.011349 | 0.1317 | 1.071176 | 0.2195 |
|  | APCD | 486 | 1 | 11 | 1.796296 | 1.51256 | 0 | 1.187587 | 0 |
|  | SFSD | 32 | 1 | 3 | 1.21875 | 1.155502 | 0.1313 | 1.054737 | 0.2195 |
| 12 | AID | 1 | 1 | 1 | 1 | 1 | 1 | 1 | 1 |
|  | APCD | 879 | 1 | 55 | 3.244596 | 2.440348 | 0 | 1.329563 | 0 |
|  | SFSD | 9 | 1 | 1 | 1 | 1 | 1 | 1 | 1 |
